# King Edward's Hospital Fund for London

**Published:** 1906-12-22

**Authors:** 


### The Hospital

A Journal of

The Medical Sciences and Hospital Administration.

Vol. XLI.—No. 1,058. SATURDAY, DECEMBER 22, 1906.

### KING EDWARD'S HOSPITAL FUND FOR LONDON.

We have it on the authority of Lord Rothschild, the Treasurer, that this Fund is in fact a millionaire to-day. Looking back to its inception, such a result of ten years' work, must seem remarkable indeed to many. What are the causes which have produced this success? The first and great cause on which the strength of the Fund rests is the principle of personal service in the days of health in the cause of the sick, which led her late Majesty Queen Victoria to select the scheme it embodies, out of the innumerable plans to commemorate her Diamond Jubilee, largely, no doubt, because King Edward VII. believes in and practises this principle. This feeling is shared by the President, the Prince of Wales, who declared on Monday "I have always felt that the King conferred a great privilege upon me when he made me President of this Fund which he had himself initiated." It is, then, a wholesome recognition of the privilege of personal service, originating in the Sovereign and spreading by conviction through all classes, from the wealthiest to the poorest, which has made the King's Fund and the League of Mercy the undoubted power for good, which they have become in a single decade of years.

Men will not give great sums of money to an object, unless they believe its principles can only radiate rapidly from a position so strengthened. So it has come to pass that financial strength and financial weakness, the peer and the poorest of the citizens, have gladly joined hands to make King Edward's Hospital Fund strong enough, to fulfil the purposes for which it was originated. In fact this Fund has received from one donor at least half a million sterling, and regular gifts of one shilling a year from tens of thousands of poorer Londoners.

To accomplish the object of raising from £100,000 to £150,000 each year to strengthen the voluntary hospitals, the original scheme aimed at promoting three sources of revenue. First from capital. This was to consist of gifts of money in large sums from the relatively few but wealthy people. This object has been fulfilled, mainly through the splendid generosity of Lord Mountstephen and Lord Strathcona, supplemented by the bequest of the late Mr. Sam Lewis of a quarter of a million, which will soon be received by the Fund. The second source of income was to be annual subscriptions and donations. This portion of the scheme still lingers and halts in its progress, a fact made apparent by the circumstance, that, the total annual subscriptions from all sources yielded £23,318 in the first year of the Fund, and that they had only increased to £36,000 at the end of the last financial year. The donations in the first year amounted to £183,478, and last year the yield from this source was but £7,811. The President, therefore, has good warrant for asking for more support in annual subscriptions as a thank-offering from the many, who have received direct or indirect benefits from the London hospitals.

The third source of revenue was to be small subscriptions of from one to ten shillings per annum, a branch of work taken in hand by the League of Mercy in 1899, which has produced in income £18,000 this year, and a total contribution after seven years' work of £79,000. It follows from these facts that, whereas the wealthy or some of them, and the poorer Londoners are doing their duty by the Fund, the great majority of Londoners who may be generally denominated as prosperous citizens, have so far failed to accept the privilege of personal service, through this Fund. We venture to hope, that, every man and woman of intelligence throughout the whole metropolis will think this matter out during the Christmas season, and resolve to give, as the Prince suggests, a thank-offering each year. Not one of them would miss a guinea per annum, to commence with the New Year, and who of them can venture to hesitate in such a cause?

The possession and command of money entails grave responsibilities upon its possessor. The Fund having become a millionaire, it is essential to secure that its administration and management shall be placed upon a permanent and strong basis. Hence we welcome the proposal to incorporate the Fund by Act of Parliament, for the statutory powers thus conferred will secure that its council and committees shall be so organised, that, their respective duties and responsibilities are more closely defined than they have hitherto been. The King's Fund rests upon the wide basis of voluntary thank-offerings from every class of His Majesty's subjects within the metropolis of the Empire. It is essential therefore that the King's Fund shall be safeguarded against any possibility of a few people ever obtaining control of this powerful and representative organisation. The real principle for its successful government must be the greatest good of the greatest number of the citizens of London. This involves the wise supervision of the administration and condition of the voluntary hospitals and kindred institutions to which grants may be made, upon a plan, which shall encourage the highest forms of management, whilst it guards against undue interference with the full liberty of those, who may be responsible for the maintenance and efficiency of individual institutions. This policy has made King Edward the most successful and popular of monarchs, and has inspired the Prince of Wales as President of the King's Fund, to make some of the most informing and helpful speeches of modern times. It will no doubt be maintained and upheld by the new statutes for the control of the business of King Edward's Hospital Fund.

